# The Multifaceted Role of SNARE Proteins in Membrane Fusion

**DOI:** 10.3389/fphys.2017.00005

**Published:** 2017-01-20

**Authors:** Jing Han, Kristyna Pluhackova, Rainer A. Böckmann

**Affiliations:** ^1^Department of Biochemistry and Molecular Biology, School of Basic Medical Sciences, Xi'an Jiaotong University Health Science CenterXi'an, China; ^2^Computational Biology, Department of Biology, Friedrich-Alexander Universität Erlangen-NürnbergErlangen, Germany

**Keywords:** SNARE, membrane fusion, synaptobrevin, syntaxin, SNAP-25, protein-lipid interactions, fusion regulation

## Abstract

Membrane fusion is a key process in all living organisms that contributes to a variety of biological processes including viral infection, cell fertilization, as well as intracellular transport, and neurotransmitter release. In particular, the various membrane-enclosed compartments in eukaryotic cells need to exchange their contents and communicate across membranes. Efficient and controllable fusion of biological membranes is known to be driven by cooperative action of SNARE proteins, which constitute the central components of the eukaryotic fusion machinery responsible for fusion of synaptic vesicles with the plasma membrane. During exocytosis, vesicle-associated v-SNARE (synaptobrevin) and target cell-associated t-SNAREs (syntaxin and SNAP-25) assemble into a core trans-SNARE complex. This complex plays a versatile role at various stages of exocytosis ranging from the priming to fusion pore formation and expansion, finally resulting in the release or exchange of the vesicle content. This review summarizes current knowledge on the intricate molecular mechanisms underlying exocytosis triggered and catalyzed by SNARE proteins. Particular attention is given to the function of the peptidic SNARE membrane anchors and the role of SNARE-lipid interactions in fusion. Moreover, the regulatory mechanisms by synaptic auxiliary proteins in SNARE-driven membrane fusion are briefly outlined.

## Introduction

**Figure 1:**
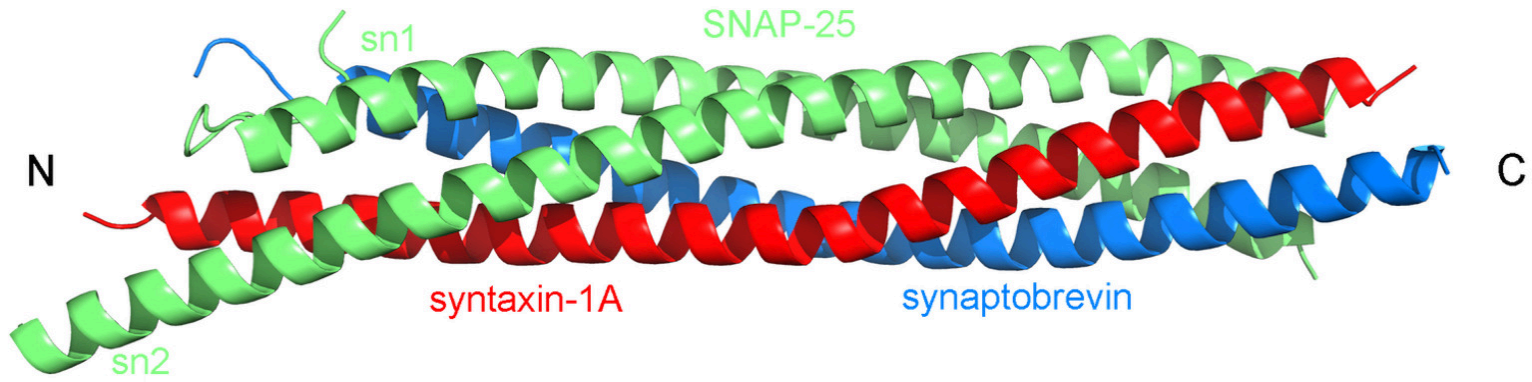
**Topology of the SNARE complex consisting of synaptobrevin (in blue), syntaxin-1A (in red), and two SNAP-25 (sn1 and sn2, both in green) proteins (PDB:1SFC, Sutton et al., 1998)**.

Biological membranes separate the cell interior from its environment and allow for the compartmentalization within the cell. They are involved in a variety of cellular events, e.g., cell signaling, exocytosis, and ion conductance. Membrane fusion is the process by which two initially separated lipid bilayers merge to form a single unity. It is a universal biological process in life, that is involved in many cellular events, e.g., in viral infection, fertilization, and intracellular trafficking. Fusion is essential for the communication between cells and between different intracellular compartments (Jahn et al., 2003; Chernomordik and Kozlov, 2005). Spontaneous membrane fusion in living orgamisms is opposed by repulsive forces between the approaching bilayers. These forces result from electrostatic repulsion of equally charged membrane surfaces and from hydration repulsion. Moreover, the lateral tension of the bilayer interface has to be overcome (Chernomordik et al., 1987; Kozlovsky et al., 2002). The energy required to overcome the energy barrier for the fusion of biological membranes is provided by specialized fusion proteins, e.g., in exocytosis the energy results from the assembly of SNARE proteins into a “rod-like” α-helical bundle, termed *trans*-SNARE complex (see Figure 1) (Jahn et al., 2003; Rizo et al., 2006; Jahn and Fasshauer, 2012).

**Figure 2:**
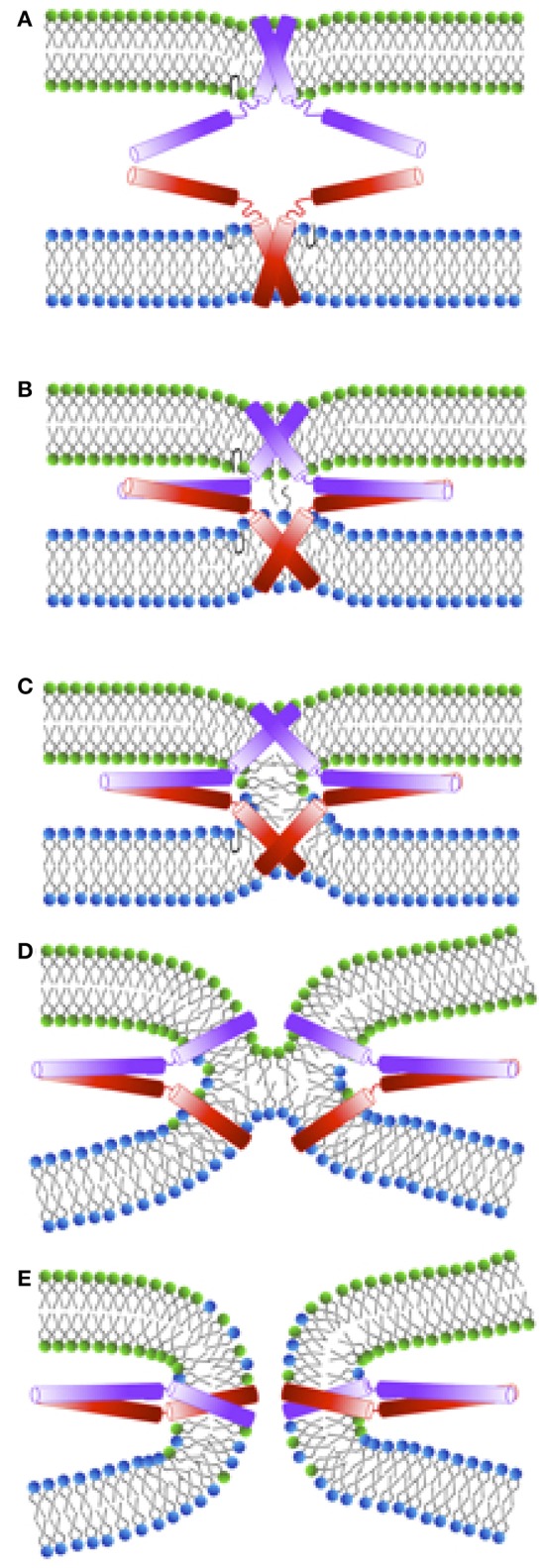
**Schematic representation of a membrane fusion process following the stalk-intermediate pathway preceded by the docking stage during exocytosis. (A)** Initial interaction between synaptobrevin (red) and syntaxin (violet) cytosolic domains. The energy released upon trans-SNARE complex formation is used to bring the membranes into close proximity (overcoming the repulsive forces between the negatively charged vesicles) and to partially dehydrate them. After SNARE complex formation the vesicles are in a “docked” state. **(B)** Upon triggering, nascent hydrophobic contacts between the approaching membranes are built between splaying lipids. **(C)** A stalk is subsequently formed and the lipids in the outer leaflets start to mix. **(D)** Stalk elongation leads to hemi-diaphragm (HD) formation and elongation. **(E)** Inner leaflets of opposing membranes begin to mix accompanied by pore formation. In the following, the pore expands until either one large vesicle is formed out of two small ones, or until all lipids from a small vesicle are fully incorporated into a planar target membrane.

Exocytosis is a complex process consisting of a several distinct stages. During “docking,” the synaptic vesicle and the plasma membrane are brought into contact (see Figure 2A). The following “priming” step renders the vesicle fusion-competent. Membrane fusion is then initiated by calcium-triggering and consists itself of several steps shown in Figures 2B–E. Although the role of SNARE complex formation in mediating exocytosis is widely accepted, the molecular mechanisms underlying the action of SNAREs at individual stages of exocytosis are still debated. Some experimental studies even suggested the necessity of SNARE only at docking and priming stages with a SNARE-independent fusion step (Tahara et al., 1998; Ungermann et al., 1998). On the other hand, a number of recent experiments shed light on the direct involvement of SNARE proteins in membrane fusion, and the formation and stabilization of various fusion intermediates were attributed to conformational changes and the dynamics of SNARE molecules.

Although the macroscopic features of exocytosis can be discerned by monitoring the content release or lipid mixing, the dynamic microscopic intermediate structures during membrane fusion are difficult or impossible to determine by experiment. However, structural and dynamic information at atomistic resolution can alternatively be obtained via molecular dynamics simulations.

This review resumes latest findings from both experiments and simulations concerning SNARE-driven fusion and exocytosis in systems ranging from model membranes to a native membrane environment by both *in vivo, in vitro*, and *in silico* investigations. Special attention is paid to the SNARE transmembrane anchors and their interactions with the host membrane. Additionally, the known regulatory mechanism by auxiliary proteins are briefly reviewed.

## 1. SNAREs in the intracellular exocytosis

The SNARE proteins constitute a large protein superfamily comprising more than 60 members in both mammalian and yeast cells. They have an evolutionarily conserved coiled-coil stretch containing 60–70 amino acids termed as SNARE motif (Fasshauer et al., 1998; Kloepper et al., 2007). The SNARE motifs in synaptobrevin and syntaxin are connected to peptidic transmembrane domains (TMDs) at the C-terminus via a short linker region. These two SNARE proteins are embedded into their respective membranes via anchoring of the TMDs. The third interaction partner, SNAP-25, consists of two SNARE motifs that are connected by a linker and attached to the plasma membrane by multiple palmitoyl tails.

### 1.1. SNARE motif

**Figure 3:**
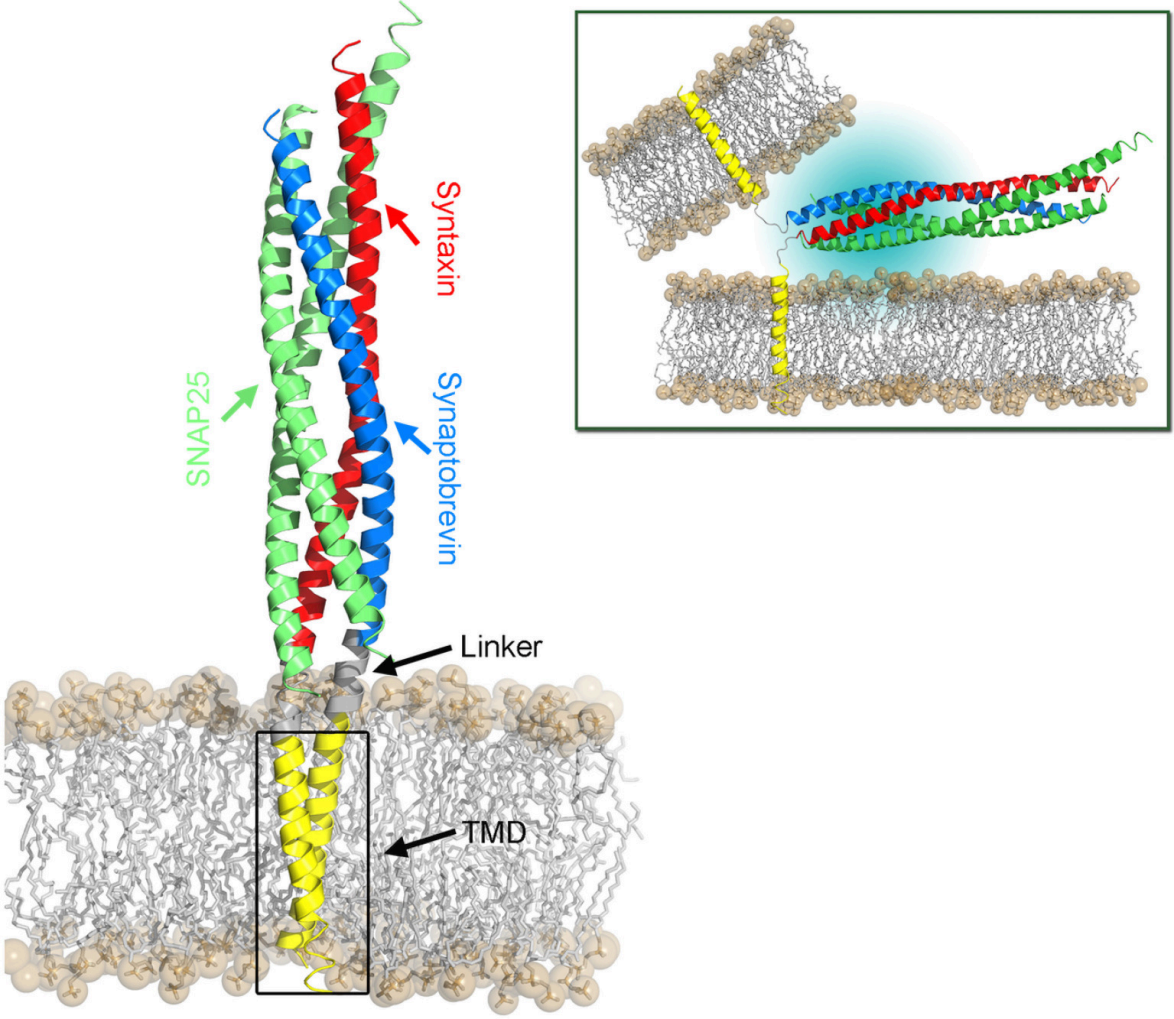
**Structural model of the SNARE complex embedded in a POPC lipid bilayer**. The *cis*-SNARE complex (PDB:3IPD) at the post-fusion stage comprises two SNAP-25, one syntaxin, and one synaptobrevin protein. The latter two SNAREs consist of a cytoplasmic domain (SNARE motifs), a short linker domain, and a transmembrane domain (TMD). The bilayer head groups are shown as spheres and hydrophobic tails as sticks. The SNARE complex in the pre-fusion stage is shown in the top right panel. Here, the TMDs of synaptobrevin and syntaxin proteins are located in their respective host membranes.

The SNARE motifs were long believed to be largely unstructured when the SNARE proteins are in monomeric forms. However, recent NMR studies on the native SNARE proteins suggested an intrinsic α-helical configuration within the SNARE motif region even in a monomeric form (Ellena et al., 2009; Liang et al., 2013). Its secondary structure is possibly modulated by membrane properties such as curvature (Liang et al., 2014) and influenced by the transmembrane domain (Han et al., 2016b). Upon exocytosis, the assembly of SNARE motifs into homo- or hetero-oligomeric bundles results in a helical configuration (Fasshauer et al., 1997; Fiebig et al., 1999; Margittai et al., 2001). A sequential assembly of SNARE motifs initiated at the N-terminal domain toward the C-terminal domain leads to the formation of tight helical bundles with extraordinary stability called the SNARE complex (Poirier et al., 1998; Sutton et al., 1998) (see Figure 3). The complex formation is accompanied by an energy release which is used to bring the membranes into close proximity (Lu et al., 2008; Hernandez et al., 2012). The *trans*-SNARE complex consists of four helices that are aligned in a parallel fashion, with synaptobrevin and syntaxin contributing one SNARE motif each whereas SNAP-25 contributes two. Progressive folding of the SNARE complex toward the transmembrane anchors results in a conversion from *trans*- to *cis*-configuration in which the SNARE proteins are fully folded and reside in the same membrane (Stein et al., 2009) (see Figure 3). This transition was proposed to be functional at the final stage of membrane fusion by facilitating the formation and expansion of the fusion pore. A corresponding mechanism could be modeled in a coarse-grained (CG) simulation study (Risselada et al., 2011). The zippering of the SNARE motif has been well characterized by two sequential zipperings from the N-terminus to the C-terminus of the SNARE motif, namely to the direction of the transmembrane segments (Li et al., 2007, 2014; Gao et al., 2012; Rizo, 2012; Min et al., 2013; Lou and Shin, 2016). This notion was confirmed by *in vitro* liposome fusion experiments in which the fusion was remarkably accelerated for a stabilized SNAP-25/syntaxin binary complex (Pobbati et al., 2006). Studies on intermediates along the ordered assembly pathway during the SNARE complex formation and on their role at distinct stages of synaptic vesicle fusion is thought to enable delineation of the specific roles of different regions of the SNARE motif. Using the high force resolution of optical tweezers, a single SNARE complex assembly could be linked to different stages of synaptic vesicle fusion (Lu et al., 2008). The spontaneous folding at the N-terminal region regulates vesicle priming by juxtaposing the membranes. The priming is followed by a fast zippering toward the C-terminal domain and finally by the fusion pore formation and expansion (Gao et al., 2012; Rizo, 2012). The zippering is controlled by regulatory proteins such as synaptotagmin in fast Ca^2+^-triggered exocytosis in neurotransmitter release. Possibly, the regulatory proteins increase the local membrane curvature (Martens et al., 2007; Hui et al., 2009; McMahon et al., 2010) and thereby destabilize the membrane. The functional importance of different regions of the SNARE motif was further indirectly confirmed by *in vitro*/*in vivo* fusion experiments using either truncated SNAREs or treatment by neurotoxin which prevents the initial zippering of SNAREs at the N-terminus (Chen et al., 2001; Siddiqui et al., 2007).

### 1.2. Linker region

**Figure 4:**
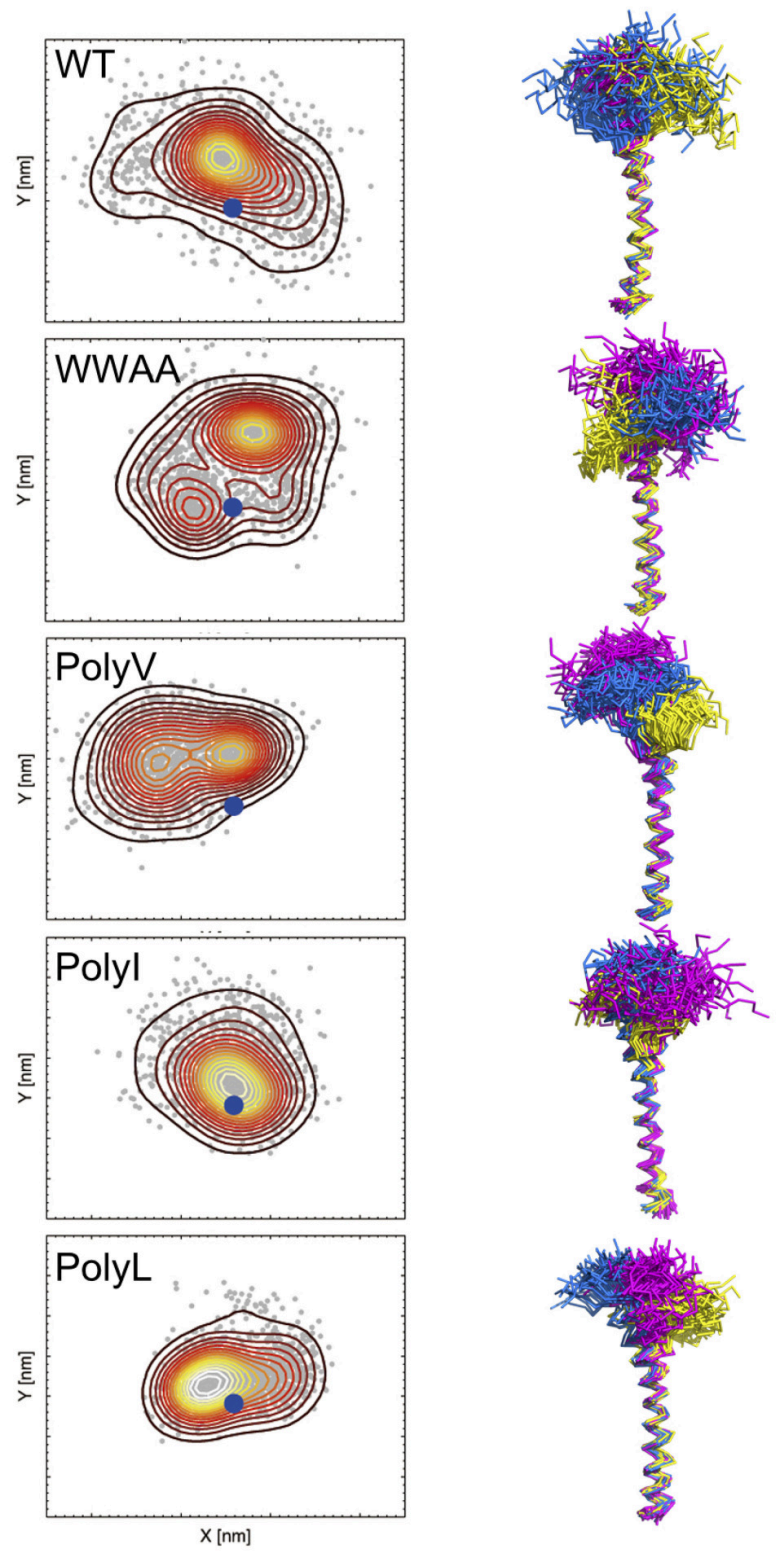
**Spatial sampling of the juxtamembrane region (JMR) in dependence of the primary TMD sequence of synaptobrevin**. The left column shows a contour density plot of the JMR configurational sampling (center of mass positions) in the bilayer plane (x-y). The blue dot marks the position of the TMD. The right column shows the side view of the sampled conformational space of the juxtamembrane region (after fitting the TMD). In the right panel sampling of three copies of the peptide (sequence as shown in Table 1) is shown in different colors, namely in yellow, blue, and violet, respectively. Samplings were recorded in the time interval from 500 to 1000 ns of atomistic simulations (see Han et al., 2016b for details).

The linker region that connects the SNARE motif and the transmembrane domain (TMD) was characterized to serve as a force-transmission machinery, which forwards the stress generated by the assembly of the cytosolic core complex toward the membrane interface and thus triggers the membrane fusion (Knecht and Grubmüller, 2003; Jahn and Scheller, 2006; Jahn and Fasshauer, 2012). The linker, in particular its positively charged residues, is also required for lipid mixing and the transition from hemifusion to full fusion (Hernandez et al., 2012). Extension of the linker region by inserting extra amino acids generally decreased the fusion efficiency. This decrease showed a length-dependent fashion and implies a tight coupling between the SNARE motif and the TMDs in the fusion machinery (Van Komen et al., 2005; Deák et al., 2006; Kesavan et al., 2007; Zhou et al., 2013). The stiffness of the linker is supposed to be important in preventing stress dissipation (Risselada et al., 2011). Moreover, the linkers of both synaptobrevin and syntaxin are fully helical in the post-fusion *cis*-SNARE complex (Stein et al., 2009). A stiff and partially structured linker was reported from a molecular dynamics study on monomeric syntaxin (Knecht and Grubmüller, 2003). However, a rigid linker might limit the reorientation of a single SNARE molecule, thus imposing an impediment for further approach of the contacting membranes in the pre-fusion state (Risselada et al., 2011; Han et al., 2016b). Moreover, the substitution of residues in the linker region with helix-breaking residues (Gly/Pro) enhancing the flexibility had little influence on the fusion efficiency (McNew et al., 1999; Van Komen et al., 2005). A growing evidence exists that both linker rigidity and flexibility are important in fusion, requiring a fine balance further regulated by the TMD as well as by other proteins and lipids (Knecht and Grubmüller, 2003; Han et al., 2016b). Thereby, an enhanced orientational sampling of the SNARE motif with minimal energy dissipation is enabled. For synaptobrevin, the secondary structure and conformational flexibility of the juxtamembrane region (JMR) were found to be directed by the sequence dependent rigidity or flexibility of the TMD (Han et al., 2016b) (see Figure 4). A cluster of charged residues in the JMR was reported to be essential for fusion capability by bridging the vesicle and plasma membranes at the onset of fusion (Williams et al., 2009). The interaction between this polybasic region and the anionic membrane surface has been shown to promote the interbilayer contact by disrupting the water ordering (Tarafdar et al., 2015). Thus, an intrinsic molecular restrain in the linker region likely affects the merging efficiency between opposing membranes, leading to an apparently decreased priming during exocytosis. Additionally, priming is as well regulated by the two tandem tryptophan residues (W89W90) in the juxtamembrane region by modifying the electrostatic potential at the membrane surface (Borisovska et al., 2012). The importance of these two residues in controlling the membrane-insertion depth of sybII and its dynamics has been emphasized in a recent NMR study (Al-Abdul-Wahid et al., 2012). The W89W90 pair at the membrane interface appears to serve as a fusion clamp in Ca^2+^-triggered fusion events (Fang et al., 2013).

### 1.3. SNARE transmembrane domain (TMD)

#### 1.3.1. Length requirements of TMD for membrane fusion

The function of SNARE TMDs in membrane fusion has been extensively investigated but is still under debate given the diverse conclusions from both experimental and simulation studies. To determine the possible roles of SNARE TMDs in membrane fusion, the transmembrane domains of SNAREs have been replaced by various lipid anchors, mutated, or partially truncated. The transition from hemifusion to fusion was reported to be blocked in SNARE-mediated proteoliposome fusion when the C-terminal half of SNARE TMD was deleted (Xu et al., 2005). Similarly, a series of truncated mutants reported for the sybII TMD was largely incompetent to support neurosecretion in PC12 cells (Fdez et al., 2010) and the lipid mixing was significantly reduced and fusion completely diminished in SNARE-mimics upon TMD shortening (Wehland et al., 2016), suggesting a stringent length requirement for the transmembrane domain.

**Table 1:** **Sequence alignment of the synaptobrevin wild type from ***Rattus norvegicus*** and its TMD mutants used in Han et al. (2016a) and based on experimental investigations of Dhara et al. (2016)**.

**Peptide**	**Sequence**
WT	74-ASQFETSAAKLKRKYWWKNLKMMIILGVICAIILIIIIVYFST-116
WWAA	74-ASQFETSAAKLKRKYAAKNLKMMIILGVICAIILIIIIVYFST-116
PolyL	74-ASQFETSAAKLKRKYWWKNLKMMLLLLLLLLLLLLLLLLYFST-116
PolyI	74-ASQFETSAAKLKRKYWWKNLKMMIIIIIIIIIIIIIIIIYFST-116
PolyV	74-ASQFETSAAKLKRKYWWKNLKMMVVVVVVVVVVVVVVVVYFST-116

The notion is further supported by the fact that vacuolar R-SNARE Nyv1p with a lipid anchor, that spans only a single leaflet, resulted in little lipid mixing in liposome fusion unless other physiologically accessory proteins were engaged (Xu et al., 2011). Moreover, the replacement of the SNARE transmembrane domain by a lipid anchor was shown to inhibit the fusion of vacuoles (Rohde et al., 2003) or of reconstituted proteoliposomes (Chang et al., 2016). In the latter study three different fusion constructs, namely Ca^2+^-triggered dense core vesicle exocytosis, spontaneous synaptic vesicle exocytosis, and Ca^2+^-synaptotagmin-enhanced SNARE-mediated liposome fusion, were tested. However, SNAREs with longer prenyl anchors spanning both leaflets were shown to be capable of driving lipid mixing to a similar extent as native SNAREs (McNew et al., 2000). Also, the TMD was recently reported to serve as a non-specific membrane-anchor in driving the vacuolar fusion implying that a lipidic anchor is sufficient for fusion (Pieren et al., 2015). In contrast, for geranylgeranylated TMDs of yeast SNAREs an inhibitory effect on exocytosis was observed. However, fusion could partially be rescued by addition of inverted cone-shaped LPC to the inner leaflet resulting in a positive curvature that promotes the fusion pore formation. This finding indicates an active role of TMD on the membrane topology (Grote et al., 2000). Additionally, lipid-anchored SNARE proteins (syntaxin and synaptobrevin) without TMDs were found to rescue the spontaneous synaptic vesicle fusion as efficiently as the TMD-anchored SNAREs at physiological conditions, albeit less efficiently for the Ca^2+^-triggered exocytosis, questioning the functional role of SNARE TMDs in exocytosis (Rizo and Xu, 2013; Zhou et al., 2013). A direct evidence for the importance of the TMD for membrane fusion comes from an *in vitro* reconstituted liposome fusion study. Therein synthetic peptides mimicking the hydrophobic cores of SNARE TMDs lacking the soluble domains were found to support membrane fusion (Langosch et al., 2001; Hofmann et al., 2006). In contrast, pure liposomes or liposomes harboring oligo-leucines showed negligible fusion activity. These studies hint to a critical sequence-dependent function of SNARE TMD in membrane fusion by facilitating both the hemifusion and complete fusion.

Taken together, under *in vitro* conditions the TMD has to span both layers to support the hemifusion to fusion transition. *In vivo*, different molecular mechanisms may likely replace (some of) the functions of the TMD anchors in membrane fusion.

#### 1.3.2. Conformational flexibility of TMD relates to the fusion capacity

It was proposed that the capability of the SNARE TMD to promote exocytosis is intimately correlated with its conformational flexibility (Dhara et al., 2016). A measure of the latter is given by its secondary structure content in solution. The wild-type SNARE TMDs (compare Table 1 for sybII TMD sequences) adopt a mixture of α-helical and β-sheet structure in solution whereas a mutant with increased helicity showed a decreased fusion activity (Langosch et al., 2001). Dhara et al. (2016) reported a clear dependency between the conformational flexibility and fusion activity for different synaptobrevin TM mutants. Likewise, a mutation that increased the stability of the TMD helix of VSV (vesicular stomatitis virus) G-protein inhibited membrane fusion (Dennison et al., 2002). The conformational flexibility of the TM helix was ascribed to the presence of β-branched residues (Ile/Val), as evidenced by the overexpression of these residues in SNARE TMD and also in viral fusion proteins (Cleverley and Lenard, 1998; Langosch et al., 2001, 2007). The fact that β-branched residues promote the flexibility of TMD was further supported by an electron spin resonance (EPR) spectroscopy study on the TMD of yeast SNARE (Sso1p), which showed an enhanced motional dynamics in the C-terminal end of TMD comprised of a stretch of valine residues (Zhang and Shin, 2006). *De-novo* design of a series of fusogenic peptides that contained a mixture of the helix-promoting residues, leucine, and the β-sheet-promoting residue, valine, showed a strong correlation between the relative ratio of valine in the TM sequence and helix flexibility. The latter can be further enhanced by the introduction of helix-breaking residues Gly/Pro (Hofmann et al., 2004). The increased conformational flexibility led to an enhanced activity of fusion peptides corroborating the notion that structural flexibility is crucial for TM peptides to be able to drive membrane fusion.

Although the TMD flexibility is endowed by structural features with reversible helix/sheet conversion in solution, it is unlikely that this transition occurs in the hydrophobic membrane interior. The flexibility is rather reflected by a transient unfolding of backbone hydrogen bonds (Langosch et al., 2001, 2007; Langosch and Arkin, 2009; Han et al., 2016b). However, it remains unclear how the flexible TMD mediates membrane fusion. It has been suggested that the backbone dynamics may disturb the structure of surrounding lipids and thus promote the lipid mixing (Langosch et al., 2007; Langosch and Arkin, 2009). This model was later supported by hydrogen/deuterium exchange analysis on the SNARE transmembrane helices in solution and in the membrane. These experiments demonstrated that the conformational dynamics of SNARE TMDs is correlated with their ability to promote lipid mixing (Stelzer et al., 2008). Alternatively, a flexible TMD may stabilize the intermediate non-lamellar structure in the late stage of fusion reaction or facilitate the formation of a fusion pore, as proposed by Dennison et al. (2002).

#### 1.3.3. The uncharged C-Terminus of SNARE TMD initiates fusion pore formation

Despite the well documented functional importance of the SNARE TMD in facilitating the formation of the stalk and hemifusion states, the role of SNARE TMDs in promoting complete fusion is still poorly understood. The transition from the hemifusion state to full fusion is initiated with the formation of a fusion pore accompanied by merging of inner leaflets from both vesicle and target membranes, followed by pore expansion and content release (Chernomordik and Kozlov, 2008; Jackson and Chapman, 2008; Rizo and Rosenmund, 2008). The formation of a fusion pore appears to be related to both the mechanical stress upon SNARE complex assembly and the displacement of the C-terminus of the transmembrane segment (Ngatchou et al., 2010; Risselada and Grubmüller, 2012).

**Figure 5:**
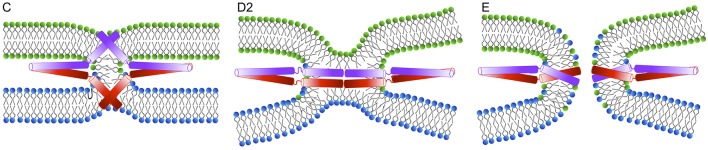
**An alternative mechanism of fusion pore formation by the insertion of the TMDs into the membrane interior as suggested by Fang and Lindau (2014)**.

The critical role of the SNARE TMD in promoting the transition from hemifusion to fusion has been demonstrated by several studies. The formation of the fusion pore was suggested to be initiated by the movement of the sybII TMD uncharged C-terminus into the membrane interior, induced by the pulling force resulting from SNARE complex zippering, as revealed by coarse-grained simulations (Lindau et al., 2012) (see Figure 5). Addition of charged residues to the C-terminus of SNARE TMD inhibited exocytosis in chromaffin and PC 12 cells (Ngatchou et al., 2010; Wehland et al., 2016), suggesting a mechanism in which the movement of the C-terminus initiates the fusion pore formation by rearranging the bilayer structure in distal leaflets (Fang and Lindau, 2014).

## 2. SNARE-SNARE interactions in exocytosis

### 2.1. SNARE organization in the prefusion state

Multimerization of SNARE complexes is essential in fast regulated exocytosis in neuronal transmission (Montecucco et al., 2005). A prerequisite for the functioning of SNARE proteins in exocytosis is the proper targeting into specific regions that define the sites for the fusion initiation (Lang et al., 2001). An extraordinary density of SNAREs is present in both synaptic vesicle and plasma membranes that is related to cluster formation (Takamori et al., 2006; Sieber et al., 2007; Bar-On et al., 2012). The t-SNARE syntaxin has been found to form dynamic clusters (Rickman et al., 2010; Bar-On et al., 2012; Ullrich et al., 2015) with increased cluster size and abundancy at the presynaptic active zone (Ullrich et al., 2015). The clustering of syntaxin is mediated by weak protein-protein interactions and is highly dynamic (Ullrich et al., 2015). It can be disrupted by the presence of as little as 1% of anionic PIP2 lipids (Murray and Tamm, 2011). The organization of SNAREs into clusters may be functional as reservoirs of SNARE molecules, allowing for rapid interactions with their cognate partners (Rickman et al., 2010; Bar-On et al., 2012). Alternatively, the multimerization of the SNARE complex was also reported to be mediated by the interaction between the SNARE motifs of SNAP25 and syntaxin via forming an ionic couple to link neighboring SNARE complexes, thus leading to a radial organization (Megighian et al., 2013). EPR and fluorescence studies showed that SNARE proteins could self-assemble through the interaction between TMDs, and the oligomeric structure could serve as a scaffolding for the formation of the trans-SNARE complex which leads to the formation of a supramolecular structure containing at least three copies of SNARE complexes (Lu et al., 2008). In addition, the accessory protein complexin has been found to be functional in mediating SNARE oligomerization at the prefusion stage (Tokumaru et al., 2001; Kümmel et al., 2011), possibly via organizing the SNARE complexes in a *zig-zag* fashion. Furthermore, the macromolecular structure resolved by electron tomography at presynaptic active fusion sites in intracelluar vesicle exocytosis has shed light on the organization of a supra-complex, which was suggested to be comprised of clustered SNAREs and other regulatory proteins like the Ca^2+^ sensor synaptotagmin (Szule et al., 2015).

### 2.2. Interactions of SNARE TMDs promote the hemifusion to fusion transition

*In vitro* fusion experiments of liposomes with reconstituted SNARE proteins of varying densities provided a deeper insight into the mechanism for the transition from hemifusion to fusion. For low surface density of SNAREs in liposomes, the hemifusion state was arrested, implying a cooperative action of SNARE proteins in promoting the hemifusion to fusion transition (Lu et al., 2005; Xu et al., 2005). The density of SNARE proteins is considered to be the most important parameter in determining not only the fusion kinetics but also the extent of liposome fusion mediated by SNAREs (Ji et al., 2010).

Along the same line, the synthetic peptides harboring the transmembrane segment of yeast vacuolar Q-SNARE Vam3p were evaluated for their fusogenic potency in proteoliposome systems. The propensity for the hemifusion transition to full fusion represented by the inner leaflet mixing increased as the surface density of the incorporated peptide increased (Hofmann et al., 2006). In addition, introduction of mutations on the interaction interface of the synthetic peptide significantly inhibited the inner leaflet mixing in liposome fusion. This defect was fully rescued to wild-type fusion efficiency when the TMDs were covalently dimerized. The full-length protein with the same mutations in the TMD also displayed a reduced content mixing in yeast vacuolar fusion (Hofmann et al., 2006), suggesting a potential function of TMD-TMD interactions in promoting the transition from hemifusion to fusion. This notion is further supported by the SNARE-induced proteoliposome fusion regulated by cholesterol under physiological conditions. The v-SNARE TMD forms an “open-scissor” dimer and its conformational change to a parallel dimer was proposed to be critical for fusion activity (Tong et al., 2009). Another study demonstrated that homodimerization of R-SNARE sec22 is critical for efficient exocytosis; This protein appears to be involved in the assembly of SNARE complex oligomers (Flanagan et al., 2015).

The formation and expansion of the fusion pore is a critical step for full fusion. The zippering of the TMDs of t- and v-SNARE proteins facilitated lipid mixing and pore nucleation and the presence of the heterodimer at the pore rim was suggested to obstruct pore resealing (Wu et al., 2016).

### 2.3. Specific dimerization of SNARE TMDs

**Figure 6:**
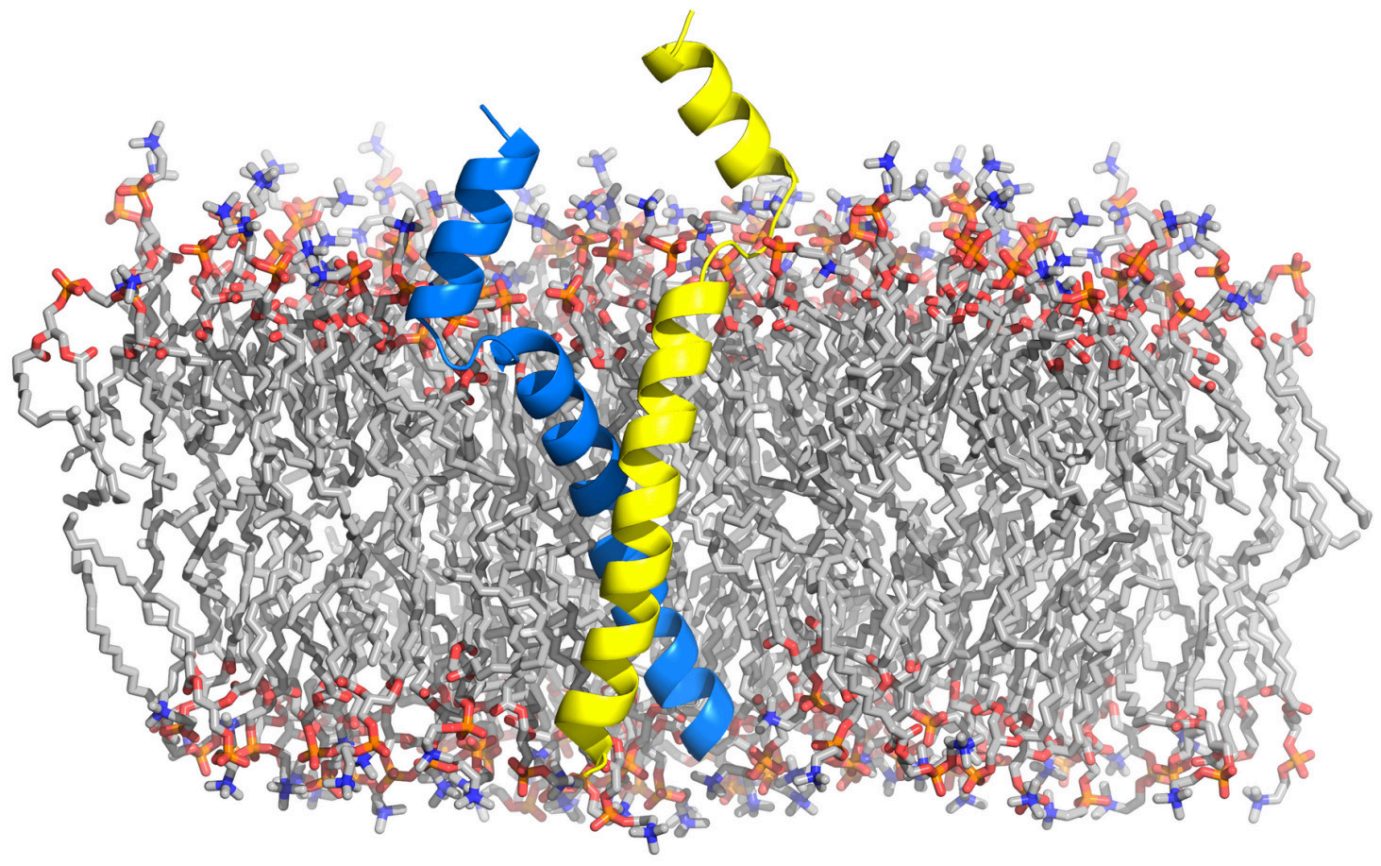
**Structural model of synaptobrevin's most abundant TMD dimer embedded in a POPC bilayer (Han et al., 2015) as obtained from a sequential multiscaling approach (Pluhackova and Böckmann, 2015)**.

As discussed above, the interactions between SNARE TMDs were found to promote the hemifusion to fusion transition. TMD-TMD interactions have previously been demonstrated to mediate the homo- and heterodimerization of SNARE proteins (Laage and Langosch, 1997; Margittai et al., 1999; Laage et al., 2000; Roy et al., 2004; Kroch and Fleming, 2006; Roy et al., 2006; Han et al., 2015). A conserved motif in the transmembrane segments was identified for the specific interaction between SNARE proteins (Laage and Langosch, 1997; Margittai et al., 1999; Laage et al., 2000). The sybII TMDs could pack tightly to form a right-handed dimer structure (Fleming and Engelman, 2001; Han et al., 2015) (see Figure 6). In a different study, the binding affinity between sybII TMDs was reported to be small and the role of dimerization in membrane fusion was challenged (Bowen et al., 2002). The association propensity of sybII actually depends on experimental conditions (Roy et al., 2004). The TMDs of syntaxin form stable homodimers with an association free energy of -3.5 kcal mol^−1^, as determined by analytical ultracentrifugation. A comparable association propensity for sybII TMD could be obtained for peptides including a single mutation at position 99 (Leu/Met) (Kroch and Fleming, 2006). In addition, it was proposed that alternative binding interfaces in the TMD of syntaxin are responsible for homo- and heterodimerization. The presence of alternative interfaces in the SNARE TMDs are implicated by their oligomerization (Laage and Langosch, 1997; Kroch and Fleming, 2006; Zhang and Shin, 2006; Han et al., 2015) and by the conversion between homo- and heterodimers of SNARE proteins. The latter is thought to be involved in the final stage of membrane fusion, leading to *cis*-SNARE complex formation (Stein et al., 2009). In line with these implications, several dimerization interfaces were recently reported based on sequential multiscaling MD simulations of the dimerization of sybII TMD (Han et al., 2015). The alternative dimerization interfaces were less populated than the main dimerization interface and dynamic interconversions between the individual interfaces were observed (Han et al., 2015). The importance of those multiple interfaces for oligomerization was confirmed in a follow-up work (Han et al., 2016a).

### 2.4. SNARE oligomerization in fast exocytosis

Despite the functional importance of protein-protein interactions in SNARE-mediated membrane fusion and the knowledge about the structure of the TMD oligomers, it still remains elusive how the transition from hemifusion to fusion is accomplished. It is well established that a single SNARE complex constitutes a minimal machinery in reconstituted proteoliposome studies, albeit with relatively slow kinetics (Weber et al., 1998; van den Bogaart et al., 2010). Fast exocytosis, that occurs at millisecond timescale during neurotransmitter release, requires cooperative action of several SNARE complexes (Montecucco et al., 2005; van den Bogaart and Jahn, 2011; Shi et al., 2012). A small number of SNARE complexes might significantly reduce the fusion rate, as demonstrated by a single vesicle fusion assay (Karatekin et al., 2010). Recent coarse-grained simulations have shed light on the cooperative effects of multiple SNARE complexes in membrane fusion. The process was remarkably accelerated when several SNARE complexes were incorporated, albeit one SNARE complex was found sufficient to drive fusion (Risselada et al., 2011). The required number of SNARE complexes for efficient fusion is also influenced by the lipid composition as bilayers containing fusion-promoting lipids such as PE/PS fuse as efficiently even with a reduced number of SNARE complexes (Domanska et al., 2010). The required number of SNARE complexes for vesicle fusion varies and depends on the physiological conditions such as the vesicle type or the timing demand (Montecucco et al., 2005; van den Bogaart and Jahn, 2011; Hernandez et al., 2014): Two SNARE complexes are necessary for fast synaptic transmission in cultured hippocampal neurons (Sinha et al., 2011), while at least three neuronal SNARE complexes are required for fast fusion exocytosis in chromaffin and PC12 cells (Hua and Scheller, 2001; Mohrmann et al., 2010). A theoretical model based on atomic force microscopy (AFM) measurements on the interaction force between SNARE proteins proposed that at least four SNARE complexes are required for fusion (Yersin et al., 2003). Using a single vesicle fusion assay, the number of SNARE complexes to trigger fusion at millisecond time scale was estimated to be eight (Domanska et al., 2009). An even higher number of 15 SNARE complexes was proposed for exocytosis using botulinum neurotoxins which specifically inactivate SNAREs (Montecucco et al., 2005).

In the post-fusion state, SNARE complexes were suggested to be arranged in a rosette-shaped multimeric bundle (Montecucco et al., 2005) or to form star-shaped oligomers (Rickman et al., 2005). It has also been suggested that SNARE complexes could be organized in a circular fashion leading to a pore-like structure when the isolated full-length SNARE proteins are reconstituted into liposomes. The size of this SNARE supercomplex depends on the vesicle diameter as detected by atomic force microscopy (AFM) and electron microscopy (Cho et al., 2005, 2011). It has been speculated that the rim of the hemifusion diaphragm is lined by the proteinaceous TMDs from several SNARE proteins which form ring-like oligomers (Chernomordik and Kozlov, 2003). An indirect evidence comes from a study in which mutation along one face of the syntaxin transmembrane helix altered the transmitter flux in PC12 cells and also the fusion pore conductance (Han et al., 2004). Consequently, a proteinaceous fusion pore model comprising at least 5-8 syntaxin TMDs was proposed. Similarly, substitution of residues close to the N-terminus of sybII TMD by bulky tryptophan residues or by charged residues also modified the fusion pore flux and conductance, implying a possible complementary pore model constituted by three or four sybII dimers (Chang et al., 2015). These ring-like arrangements were, however, shown to be incompatible with the tight vesicle docking observed in a reconstituted membrane fusion system (Hernandez et al., 2012).

**Figure 7:**
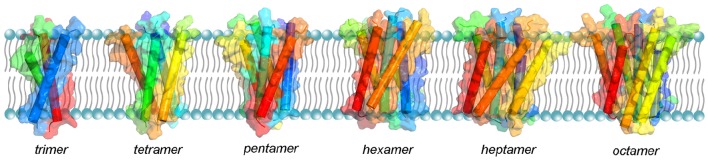
**Structural models of sybII oligomers containing 3–8 copies of transmembrane domains (left to right)**. The sybII oligomer structures were obtained either from self-assembly CG-MD simulations (trimer and tetramer) or from manual built (pentamer to octamer), see Han et al. (2016a).

The oligomerization mechanism and the shape of the sybII TMD oligomers (see Figure 7) was presented in a recent MD simulation study which shed light on the stepwise self-assembly of sybII TMDs into oligomers (Han et al., 2016a). The oligomers were discovered to be either compact or linear, the former being more stable. The form of the oligomers contradicts the pore-hypothesis based on the mutagenesis data of Chang et al. (2015). Namely, the residues that were suggested to point to the lumen of the proteinaceous pore are positioned on the outside of the oligomer providing a binding interface for other sybII TMDs (Han et al., 2016a). Moreover, the tryptophanes in positions 89 and 90 were found to endow synaptobrevin oligomers with increased dynamics and reduced compactness. Thereby, the oligomers likely provide an enlarged accessibility to its cognate partner upon SNARE complex formation.

## 3. Importance of protein-lipid interactions for membrane fusion

The large structural rearrangements of membranes during fusion are typically driven by fusion peptides and further modulated in particular by the lipid composition (Aeffner et al., 2012). Upon first contact of the approaching membranes their surfaces have to get dehydrated and the lateral tension of the bilayer interface has to be overcome (Chernomordik et al., 1987; Kozlovsky et al., 2002). The membrane composition influences the pathway preference and the fusion rate while the fusion proteins reduce the energy barrier for the fusion process via specific protein-protein and protein-lipid interactions (Chernomordik and Kozlov, 2003; Jahn et al., 2003). The fusion propensity of lipid bilayers depends on their lipid composition and mainly on the intrinsic lipid geometry (Chernomordik and Kozlov, 2003). While the hemifusion is inhibited by inverted cone-shaped LPCs and promoted by cone-shaped PEs, the pore formation is facilitated by LPCs and inhibited by PEs positioned in the distal leaflet of fusing membranes (Chernomordik and Kozlov, 2008).

**Figure 8:**
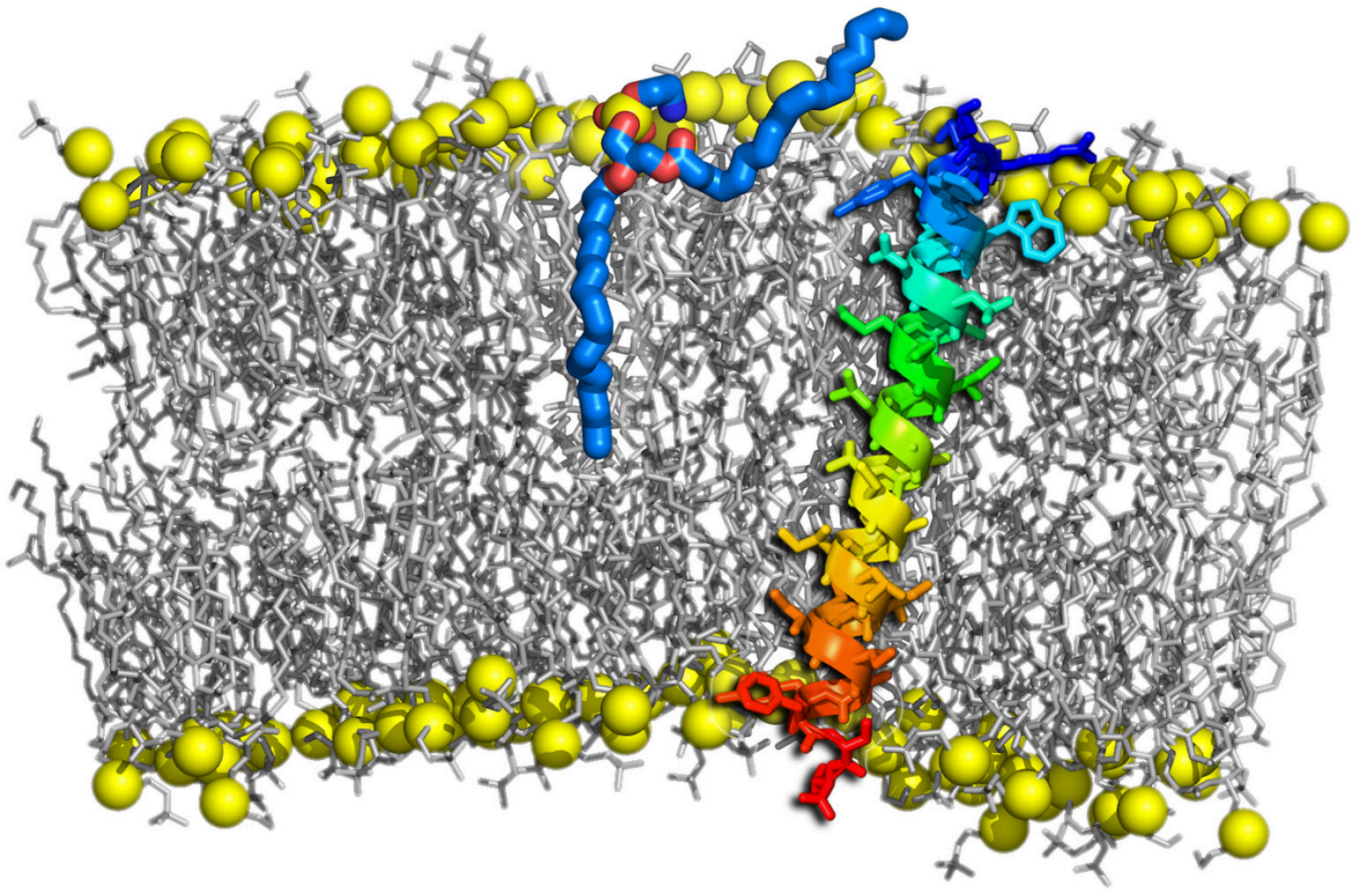
**Lipid tail protrusion in a curved POPC membrane evoked by a sybII TMD peptide**. Lipids are shown as gray sticks with phosphates highlighted as yellow spheres. The peptide is shown as a rainbow-colored cartoon and sticks. The protruding lipid is highlighted as thick sticks with carbons colored blue and oxygens red.

A wealth of evidence from simulations has demonstrated that the critical step at the onset of fusion are hydrophobic contacts between the approaching membranes, formed e.g., by protruding lipids (see Figure 8). The initial lipid splaying is followed by the formation of a lipid tail stalk (Kinnunen, 1992; Holopainen et al., 1999; Marrink and Mark, 2003; Stevens et al., 2003; Kasson et al., 2010; Smirnova et al., 2010).

The lipid protrusion probability and partial dehydration of the outer membrane leaflet were shown to be enhanced in presence of a shortened synaptobrevin construct containing the TMD, the linker, and the juxtamembrane domain carrying positively charged residues (Han et al., 2016b). These results hint to an additional role of SNARE peptides in destabilizing its immediate lipid environment. Also the α-helical SNARE TM anchors were shown to be associated with bilayer destabilization (Risselada et al., 2011) and lipid mixing, discussed in the context of their oblique orientation in membranes. An increased number of lipid acyl chain protrusion events was recently also observed in curved membranes (Rabe et al., 2016; Tahir et al., 2016). Additionally, SNARE complexes were reported to induce local membrane protrusions in the target membrane (Bharat et al., 2014). The characteristics of curved membranes are of inherent interest for membrane fusion as both synaptic vesicles or virions (viral particles) are highly curved and since fusion intermediates involve a huge curvature. In general, the fusion rate increases with a decreasing vesicle radius (Bharat et al., 2014). Multiple reasons regarding how highly curved membranes facilitate the fusion process have been suggested, including smaller surface tension of larger vesicles, the relief of lipid stress in highly curved regions upon transition to the hemifused state (McMahon et al., 2010; Kawamoto et al., 2015), and a closer contact of fusing membranes due to membrane buckling (McMahon et al., 2010).

Both, synaptic vesicles and plasma membranes, contain negatively charged lipids in their outer and inner layers, respectively. In the prefusion state, negatively charged lipids cause electrostatic repulsion between the membranes thus preventing spontaneous fusion. This allows an exact timing of membrane fusion by addition of bivalent (typically calcium) ions. These do not only shield the lipids' negative charges but may also bridge the opposing membranes by binding two negatively charged lipids to one bivalent ion (Böckmann and Grubmüller, 2004; Pannuzzo et al., 2014). Investigations of the interactions between anionic PIP2 lipids and syntaxin were found to drastically depend on Ca^2+^. In presence of Ca^2+^, PIP2 lipids were shown to condense syntaxins together into large agglomerates (van den Bogaart et al., 2011), whereby Ca^2+^ bridges multiple syntaxin/PIP complexes (Milovanovic et al., 2016). In absence of Ca^2+^ the presence of as little as 1% PIP2 (or more than 20% PS lipids) was shown to cause disaggregation of cholesterol-dependent syntaxin clusters (Murray and Tamm, 2009). PIP2 is also known to enhance the response speed of synaptotagmin to Ca^2+^ (Bai et al., 2004) which is due to PIP2-mediated interactions either between syntaxin and synaptotagmin (Honigmann et al., 2013) or between SNAREpin and synaptotagmin (Kim et al., 2012). PS lipids were shown to mediate the interaction between complexin and the membrane in the docking state (Diao et al., 2013). Another negatively charged lipid that was found to modulate the action of SNAREs is the fusogenic phosphatidic acid (PA). If the binding of PA to a specific lipid-binding domain on syntaxin-1A is disrupted the evoked secretion is progressively reduced (Lam et al., 2008).

Moreover, cholesterol was discovered to play multiple roles during membrane fusion (Yang et al., 2016), and the exocytosis is largely decreased upon depletion of cholesterol. Cholesterol alters the lifetime of membrane fusion intermediates, it changes the membrane curvature and bending modulus, and additionally directly interacts with fusion proteins resulting in their altered distribution and modified membrane penetration depth. SNAREs were found to be either concentrated in cholesterol-dependent clusters that define docking and fusion sites for exocytosis (Lang et al., 2001; Murray and Tamm, 2009, 2011) or to be associated with the lipid raft domain (rich in cholesterol and saturated lipids) in alveolar type II cells (Chintagari et al., 2006). In giant unilamellar vesicles (GUVs), on the other hand, SNARE proteins showed preference for the disordered phase (rich in unsaturated lipids and depleted of cholesterol) (Bacia et al., 2004). Also the conformation of the synaptobrevin dimer was shown to depend on the presence of membrane cholesterol (Tong et al., 2009). A mechanistic explanation common to all those observations could be the segregation of the SNAREs by hydrophobic mismatch into domains of optimal membrane thickness (Milovanovic et al., 2015).

There is growing evidence that not only membrane-attached SNARE proteins interact with lipids and thus stimulate fusion, but that also exocytosis regulatory proteins attach to lipid bilayers. The amphiphatic C-terminal helix of complexin has been shown to bind to membranes and thus affect lipid mixing (Seiler et al., 2009). The vesicle-docking ability of complexin was moreover shown to depend on the presence of PS lipids in the membrane (Diao et al., 2013). Also the membrane binding of synaptotagmin is expected to be important for exocytosis (Lynch et al., 2008). Moreover, upon interaction with the membrane α-synuclein can either inhibit vesicle fusion (DeWitt and Rhoades, 2013) or after assembly into oligomers promote SNARE complex formation (Burré et al., 2014). The full association of homotypic fusion and protein sorting (HOPS) complex and Sec19p with proteoliposomes in the fusion of yeast vacuoles requires the presence of PA in the membrane (Mima and Wickner, 2009). In the same work, both PA and PE were shown to be essential for fusion as well as for SNARE complex assembly.

In conclusion, lipids regulate all steps of neuronal exocytosis (Ammar et al., 2013). Different lipids influence vesicle priming, the establishment of the nascent contact, the transition to hemifusion and further to the full fusion state, as well as the fusion pore opening.

## 4. Regulatory proteins in SNARE-mediated exocytosis

Apart from the SNARE complex that is characterized as the energy machinery in intracellular exocytosis, a set of regulatory proteins such as Munc18-1 (SM), synaptotagmin, and complexin in eukaryotic cells have been demonstrated to be essential for exocytosis as well. These regulatory proteins enable the exocytosis to be precisely regulated in living cells in space and time upon signal arrival, especially for Ca^2+^-triggered neurotransmitter release. Moreover, compartment-specific multisubunit tethering complexes (MTCs) regulate SNARE complex assembly and exocytosis and are responsible for trafficking to proper target compartments (Dubuke and Munson, 2016).

Munc18-1 was found to bind directly to syntaxin in closed-configuration, thus regulating the availability of syntaxin for SNARE complex assembly (Hata et al., 1993; Dulubova et al., 1999; Gerber et al., 2008; Dawidowski and Cafiso, 2016). This interaction was found to be stabilized by the H_*abc*_ domain of syntaxin (Zhou et al., 2012). Moreover, after a conformational change in domain 3a of Munc18-1 the interactions of helix12 with v-SNARE drive the SNAREpin assembly (Parisotto et al., 2014) and vesicle priming (Munch et al., 2016). The N-terminal domain of syntaxin-1 is regulated by Munc18-1 in its two different conformational states, which impose spatially distinct regulatory mechanisms, either compensating or inhibiting the active state in syntaxin trafficking (Park et al., 2016). It is intriguing that Munc18-1 is also intimately related to the transport of syntaxin to target compartments (Rowe et al., 2001).

Complexin is considered to be a specific ligand that binds to the central core domain of the SNARE complex and acts as a force clamp that controls the transfer of the force generated by SNARE complex assembly to the fusing membranes (Pabst et al., 2000; Chen et al., 2002; Maximov et al., 2009; Trimbuch and Rosenmund, 2016). Complexin was recently reported to aid the spatial organization of the SNARE complex in the pre-fusion stage (Kümmel et al., 2011) and to inrease the pool of arrested docked synaptic vesicles thus synchronizing fusion (Malsam et al., 2012). To do so, complexin's C-terminus binds to the membrane containing PS lipids, while the core domain of complexin-1 attaches to the SNARE complex (Diao et al., 2013). The fusion-inhibition effect of complexin was recently assigned to the positioning of the complexin accessory helix between the vesicle and plasma membranes rather than as earlier supposed in between SNARE proteins (Trimbuch et al., 2014). Moreover, complexin has to undergo a conformational change from an open to a close conformation so that another player, synaptotagmin, can trigger fusion upon Ca^2+^ stimuli (Krishnakumar et al., 2011).

**Figure 9:**
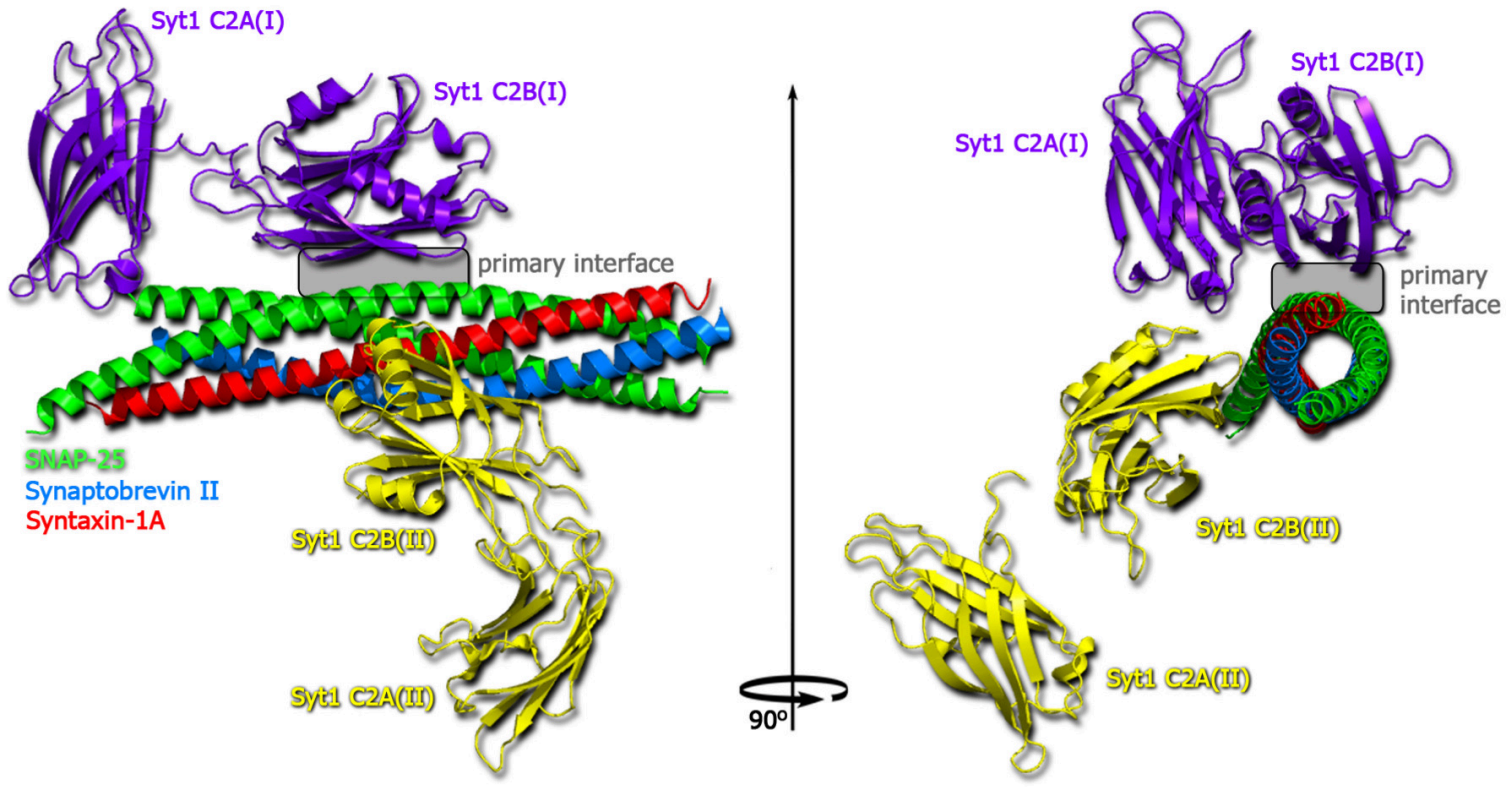
**Structure of the Ca^**2+**^-bound synaptotagmin 1-SNARE complex (Zhou et al., 2015)**. SNAP25 shown in green, synaptobrevin in blue, syntaxin-1A in red, one copy of synaptotagmin-1 in violet and one in yellow.

For synaptotagmin, diverse functions in regulating the SNARE-mediated exocytosis have been demonstrated. The crystal structures of Ca^2+^- and Mg^2+^-bound complexes of the neuronal SNARE complex and synaptotagmin-1 (see Figure 9) revealed several interaction interfaces, among those the strongly conserved and Ca^2+^-independent interface. The latter was suggested to form before Ca^2+^ triggering, to shift upon the triggering and to promote the interactions of synaptotagmin-1 with the plasma membrane (Zhou et al., 2015). In Ca^2+^-independent manner synaptotagmin-1 mediates vesicle docking (Malsam et al., 2012). Moreover, synaptotagmin is a Ca^2+^ sensor that consists of two C_2_ domains (C_2_A and C_2_B) connected by a flexible loop. These two domains were proposed to bind and insert into the membrane upon Ca^2+^ influx, creating a local curvature that significantly decreases the energy barrier for fusion pore formation (Martens et al., 2007; Hui et al., 2009; McMahon et al., 2010; Paddock et al., 2011; Krishnakumar et al., 2013). On the other side, the binding of synaptotagmin to the SNARE complex is thought to release the force clamp by displacing complexin, thus facilitating the force transfer to the apposing membranes in a Ca^2+^-dependent manner (Tang et al., 2006). Indeed, synaptotagmin acts mainly via interactions with both membranes and also with the SNARE complex. This synergistic effect facilitates the fusion pore formation and expansion upon Ca^2+^ binding (Lynch et al., 2008). In addition, synaptotagmin has been found to form ring-like oligomers (Wang et al., 2014), which are reminiscent of the oligomeric organization of the SNARE complex. Possibly, a concerted action of multiple SNARE complexes with bound synaptotagmin triggers the Ca^2+^-dependent fusion reaction in ultrafast speed, due to additive local membrane bending induced by synaptotagmin (McMahon et al., 2010).

For more information on SNARE assembly and disassembly aided by regulatory and tethering proteins see Baker and Hughson (2016) and Ryu et al. (2016). Despite the considerable progress in understanding the intracellular fusion events which involve a variety of proteins apart from the core SNARE complex, a unified picture describing the regulatory network at different levels in SNARE-mediated vesicle fusion is still missing.

## Conclusions

Membrane fusion, controlled and driven by a complex machinery of fusion and regulatory proteins, is an essential process in all living organisms. The individual proteins differ among various organisms, and even among their cells and organelles and they also depend on transported substances. Although these different proteins vary subtly in the way how they catalyze fusion, their pivotal roles stay the same.

Among all processes in which membrane fusion plays a pivotal role, exocytosis is studied most intensely and a wealth of information has been accumulated about the delicate mechanisms that regulate this process in a timely and coordinated fashion. In particular, the role of many proteins in the fusion step was addressed. Recently, research on exocytosis focused in particular on the role of membrane lipid composition, of specific protein-lipid interactions, and on the importance of suprastructures and membrane architecture. Also, multiple regulatory mechanisms by auxiliary proteins were elucidated.

Nevertheless, our understanding of fusion at the molecular scale is still limited. However, the combination of experimental techniques with MD simulations exhibits a promising avenue to uncover the subtle interplay between fusion proteins and membranes at atomistic resolution. Future studies will aim to further close the gap in spatial and temporal resolution between experiments such as electron tomography and simulation. Additionally, approaches allowing to characterize fusion pore properties using defined membrane compositions e.g., in a microfluidic setup (Vargas et al., 2014) will push our understanding of the intricate balance of protein-lipid interaction in fusion.

## Author contributions

JH, KP, and RB developed the concept of this review and wrote the manuscript.

### Conflict of interest statement

The authors declare that the research was conducted in the absence of any commercial or financial relationships that could be construed as a potential conflict of interest.
